# FRC-QE: a robust and comparable 3D microscopy image quality metric for cleared organoids

**DOI:** 10.1093/bioinformatics/btab160

**Published:** 2021-03-08

**Authors:** Friedrich Preusser, Natália dos Santos, Jörg Contzen, Harald Stachelscheid, Érico Tosoni Costa, Philipp Mergenthaler, Stephan Preibisch

**Affiliations:** Berlin Institute for Medical Systems Biology (BIMSB), Max Delbrück Center for Molecular Medicine in the Helmholtz Association (MDC), Berlin 10115, Germany; Molecular Oncology Center, Hospital Sirio-Libanese, São Paulo, SP 01308-050, Brazil; Department of Experimental Neurology, Center for Stroke Research Berlin, Charité – Universitätsmedizin Berlin, Berlin 10117, Germany; Stem Cell Core Facility, Berlin Institute of Health at Charité – Universitätsmedizin Berlin, Berlin 13353, Germany; Molecular Oncology Center, Hospital Sirio-Libanese, São Paulo, SP 01308-050, Brazil; Department of Experimental Neurology, Center for Stroke Research Berlin, Charité – Universitätsmedizin Berlin, Berlin 10117, Germany; Department of Neurology, Charité – Universitätsmedizin Berlin, Berlin 10117, Germany; BIH Academy, Berlin Institute of Health at Charité –Universitätsmedizin Berlin, Berlin 10117, Germany; Berlin Institute for Medical Systems Biology (BIMSB), Max Delbrück Center for Molecular Medicine in the Helmholtz Association (MDC), Berlin 10115, Germany; Janelia Research Campus, Howard Hughes Medical Institute, Ashburn, VA 20147, USA

## Abstract

**Summary:**

Here, we propose Fourier ring correlation-based quality estimation (FRC-QE) as a new metric for automated image quality estimation in 3D fluorescence microscopy acquisitions of cleared organoids that yields comparable measurements across experimental replicates, clearing protocols and works for different microscopy modalities.

**Availability and implementation:**

FRC-QE is written in ImgLib2/Java and provided as an easy-to-use and macro-scriptable plugin for Fiji. Code, documentation, sample images and further information can be found under https://github.com/PreibischLab/FRC-QE.

**Supplementary information:**

Supplementary data are available at *Bioinformatics* online.

## 1 Introduction

**Fig. 1.**
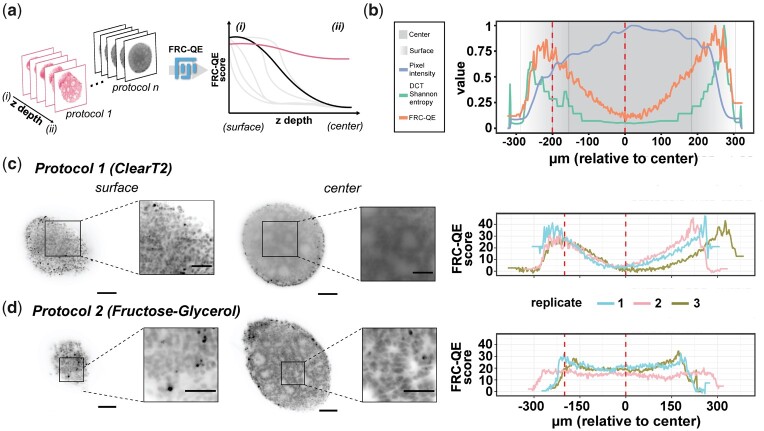
Fourier ring correlation quality estimate (FRC-QE) for assessing clearing efficiency. (**a**) FRC-QE is designed to automatically quantify clearing efficiency across multiple protocols and is provided as a Fiji plug-in. (**b**) Comparison of image quality metrics for an insufficiently cleared organoid after light-sheet multi-view reconstruction. See Supplementary Figure S7 for a detailed comparison. (**c,d**) Image quality estimates for two different protocols with three replicates each, all imaged with light-sheet microscopy and multi-view reconstructed. Replicate 1 (light blue) always corresponds to the example images shown in the left panel. Dotted red lines correspond to example slices at the surface and within the center of the organoid. Scale bars correspond to 100 and 50 μm for large panels and inlets, respectively

Three-dimensional (3D) organoids are a powerful tool for studying cellular processes in tissue-like structures, enabling in vitro experiments in an organ-specific context (Schutgens and Clevers, 2020). However, although it is essential for interpreting experiments, it remains challenging to capture cellular structures of entire organoids using 3D fluorescence microscopy due to the organoid’s dense structure and opacity. Optical clearing methods (Spalteholz 1914; Ueda *et al.*, 2020) provide a solution for fixed organoids. Nevertheless, optimizing clearing protocols for a given sample type and staining is challenging due to the plethora of available clearing and staining protocols. Importantly, quantitative measures for assessing image quality across cleared fluorescent samples are missing, making the process of identifying the best-suited protocol laborious and biased to the human observer. To fill this gap, we propose Fourier ring correlation quality estimation (FRC-QE) that provides a robust readout of image quality for 3D fluorescent microscopy samples (Fig. 1a).

## 2 Method and implementation

Fourier ring correlation (FRC) relies on two independent realizations of the same signal to measure image resolution in frequency space and was used for both electron (Heel, 1987; Saxton and Baumeister, 1982) and superresolution fluorescence microscopy (Banterle *et al.*, 2013; Koho *et al.*, 2019; Nieuwenhuizen *et al.*, 2013). We previously extended FRC to standard 3D fluorescence microscopy (Hörl *et al.*, 2019) by approximating the necessary independent observations from subsequent slices in the image stack of the same object combined with normalization to more distant slices to suppress artifacts, which was used for qualitative visualization of image quality. Here, we show that it can be used as a quantitative measurement across experiments by further adjusting (see Supplementary Materials and Methods and Supplementary Fig. S1) and validating this concept as a non-machine learning based method for no-reference image quality assessment (NR-IQA), which we call FRC-QE. Importantly, FRC-QE is designed specifically for 3D fluorescence microscopy and its score depends on the area and block size in which it is computed, the z-spacing, type of image content (e.g. nuclear stain) and the point spread function (PSF). These parameters should therefore be held constant during a series of comparisons. It is also important to note that the FRC-QE score represents arbitrary numbers that do not directly relate to an actual measurement of image resolution, but only allow for a relative comparison. FRC-QE is implemented in ImgLib2 (Pietzsch *et al.*, 2012) and the core algorithm scales to terabyte-sized datasets (Hörl *et al.*, 2019), while we also make it available to users as a macro-scriptable Fiji (Schindelin *et al.*, 2012) plugin.

## 3 Validation and results

To validate FRC-QE, we generated human induced pluripotent stem-cell (hiPSC)-derived cerebral organoids of a defined size (∼600 µm) that were stained with the nuclear dye Draq5 and subsequently chemically fixed. We chose three straight-forward to implement clearing methods (Dekkers *et al.*, 2019; Hama *et al.*, 2011; Kuwajima *et al.*, 2013) as proof of concept and applied them to our samples (Supplementary Fig. S2). Cleared organoids were imaged by multi-view light-sheet microscopy (Huisken *et al.*, 2004) from opposite acquisition angles (0° and 180°) with dual-illumination (left and right sided light-sheet illumination) and reconstructed computationally (Hörl *et al.*, 2019) to significantly increase the volume of the sample imaged with high quality (Preibisch *et al.*, 2010; Swoger *et al.*, 2007). Due to the multi-view acquisition, image quality at the edges is expected to be higher than in the center of the organoid.

We first compared FRC-QE to the NR-IQA method DCT Shannon entropy, which was previously used in the context of automated microscopy (He and Huisken, 2020; Royer *et al.*, 2016), and to plain image intensity that is commonly used to assess clearing quality (Kim *et al.*, 2018; Matryba *et al.*, 2019; Wan *et al.*, 2018). FRC-QE and DCT Shannon recapitulate image quality throughout an organoid sample, while image intensity actually increases as quality decreases (Fig. 1b and Supplementary Figs S3–S5).

We further validated FRC-QE for different microscopy modalities comparing Fructose-Glycerol-cleared organoids to uncleared organoids using a spinning-disk confocal microscope, where FRC-QE recapitulated differences in image quality across image stacks and protocols (Supplemental Fig. S6).

Next, we used FRC-QE to identify the clearing protocol yielding the best image quality. Across all protocols nuclear structures can be easily visually identified at the surface of organoids. However, image quality differs at the center, with ClearT2 and ScaleA2 protocols resulting in blurred objects compared to the Fructose-Glycerol protocol (Fig. 1c,d and Supplementary Fig. S7), indicating differences in clearing efficiency between protocols as we performed them. While DCT Shannon entropy faithfully captured relative differences in clearing efficiency in one organoid (Fig. 1b), it is not suited for comparison between samples since it does not recapitulate the visually apparent differences in clearing efficiency that we observe in between protocols. In contrast, FRC-QE accurately recapitulates these differences (Fig. 1c,d and Supplementary Fig. S7) and shows that only Fructose-Glycerol-cleared samples retain constant quality throughout entire volumes of cleared organoids.

We additionally compared FRC-QE to the established machine learning based NR-IQA algorithm BRISQUE (Supplementary Fig. S8). However, machine learning algorithms have a training phase in which they learn important characteristics about the images. These data are usually not available for biological images that differ substantially depending on the sample and staining used. Therefore, new training data needs to be created for a new type of experiment, which is often infeasible, or training is performed on natural images instead. Overall, for our data a pre-trained BRISQUE performed similarly to FRC-QE, but locally shows unexpected behavior for certain images presumably due to training on a different type of sample image. In summary, an engineered metric like FRC-QE is expected to perform more predictably in previously unseen samples and is, we believe, well-suited for biological images.

## 4 Conclusion

We introduce FRC-QE, implemented in ImgLib2 (Pietzsch *et al.*, 2012) and provided as Fiji (Schindelin *et al.*, 2012) plugin, as a new non-machine learning based NR-IQA metric to automatically assess clearing efficiency from 3D fluorescence microscopy images. FRC-QE can be applied to image data from different microscopy modalities, is comparable across protocols, and can therefore be used to identify the most suitable clearing protocol, which is often the one that achieves the necessary image quality to gain a certain insight given the lowest effort and cost. Overall, we believe that image quality estimation using FRC-QE will facilitate and significantly ease the process of choosing the right clearing protocol for a given biological sample. Furthermore, FRC-QE represents a promising approach for other automated image quality tasks in fluorescence microscopy.

## Supplementary Material

btab160_Supplementary_Data
