# Supplementary material for: FRC-QE: a robust and comparable 3D microscopy image quality metric for cleared organoids
Source: Bioinformatics. 2021 Mar 8;37(18):3088–90. doi: 10.1093/bioinformatics/btab160 (PMC8479654; doi:10.1093/bioinformatics/btab160)
Supplement: btab160_Supplementary_Data [file btab160_supplementary_data.zip › FRC_AN_Supplementals_final.pdf]

FRC-QE: A robust and comparable 3D microscopy image quality metric for cleared organoids

Supplementary Data

Supplementary Figure S1: *Overview of the FRC-QE implementation.*
Supplementary Figure S2: *Overview scheme comparing tested clearing protocols.*
Supplementary Figure S3: *Quantification of the image quality throughout the organoid.*
Supplementary Figure S4: *Multi-view quality estimation - dual illumination.*
Supplementary Figure S5: *Multi-view quality estimation - different angles.*
Supplementary Figure S6: *FRC-QE for spinning-disk confocal microscopy data.*
Supplementary Figure S7: *Comparing image quality across protocols using FRC-QE.*
Supplementary Figure S8: *Comparing FRC-QE with BRISQUE.*

Supplementary Table 1: *Imaging parameters for light-sheet imaging*

Supplementary Material and Methods

Supplementary Figures

[Supplementary Figure S1]

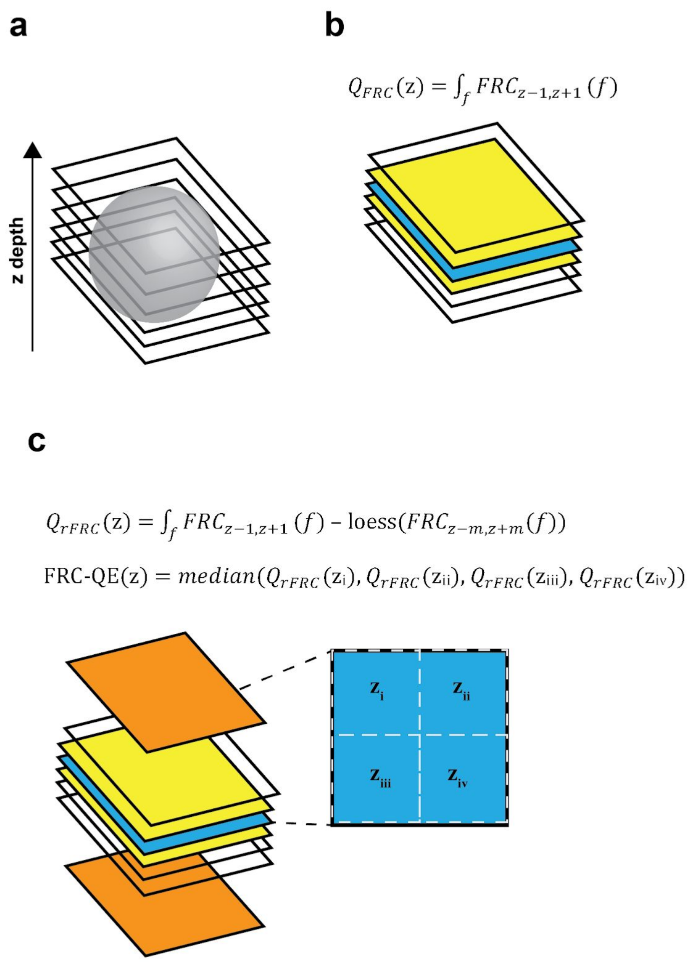

Supplementary Figure S1: Overview of the FRC-QE implementation. (a) For three-dimensional imaging, we image an object (e.g. an organoid) by acquiring several adjacent image planes. (b) FRC describes the per-spatial-frequency *(f)* correlation between two independent realizations. For each plane we correlate its adjacent planes (*z-1* and *z+1*) taking advantage of the fact that they contain very similar information due to the axial extent of the PSF. (c) To exclude nonspecific patterned noise (e.g. camera noise), a smoothed baseline FRC of planes *m* slices away of *z* is subtracted from averaged correlation scores between adjacent planes. To further reduce the influence of imaging artifacts (e.g. bright dots) on the FRC-QE, the metric can be calculated blockwise (e.g. into 4 equally sized blocks spanning the field of view) and the final FRC score per slice is calculated as the median of all blocks.

[Supplementary Figure S2]

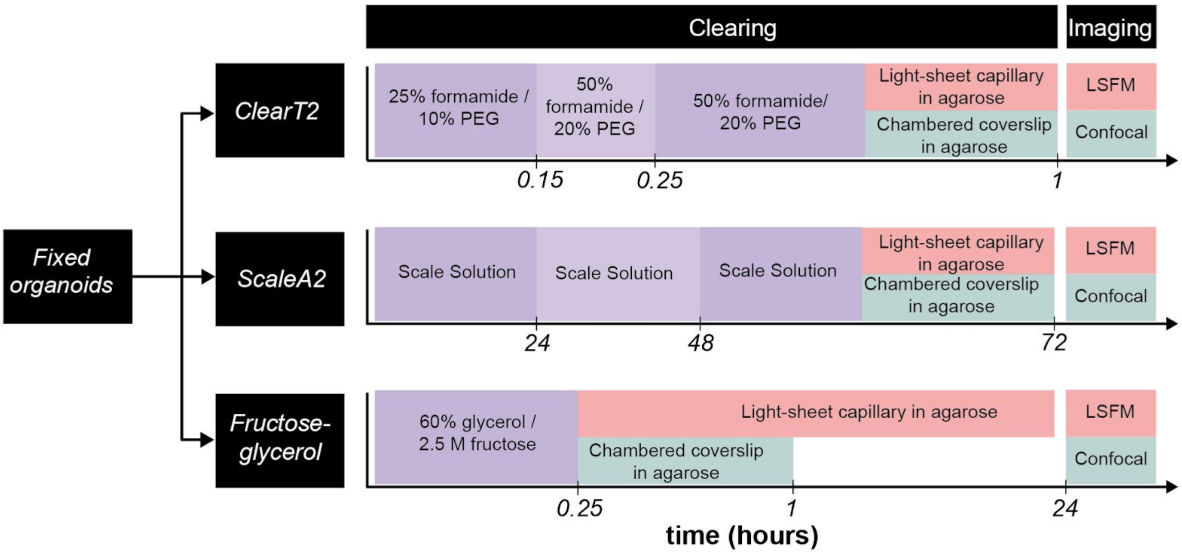

Supplementary Figure S2: Overview scheme comparing tested clearing protocols and respective experimental timing (not to scale).

[Supplementary Figure S3]

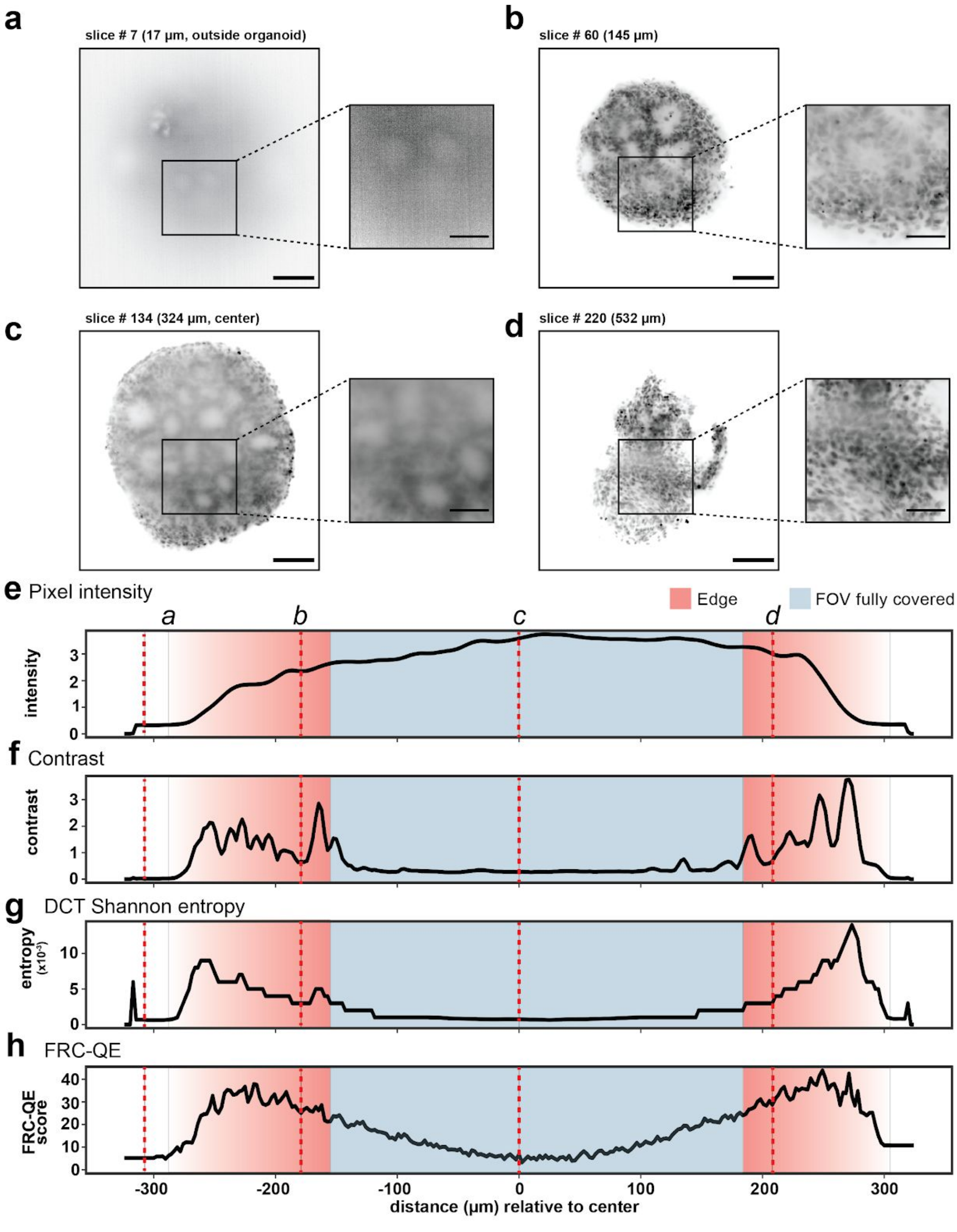

Supplementary Figure S3: Quantification of the image quality throughout the organoid. (a-d) Optical sections throughout an insufficiently cleared organoid, stained with Draq5 and reconstructed from a multi-view light-sheet acquisition. Image resolution decreases towards the middle of the organoid as seen in (c). (e-h) Different quantification modalities to assess image quality of the same organoid depicted above. Dotted red lines correspond to the corresponding panels above as indicated. Scale bars correspond to 100 µm and 50 µm for large panels and inlets, respectively. Brightness and contrast was adjusted individually for the example images.

[Supplementary Figure S4]

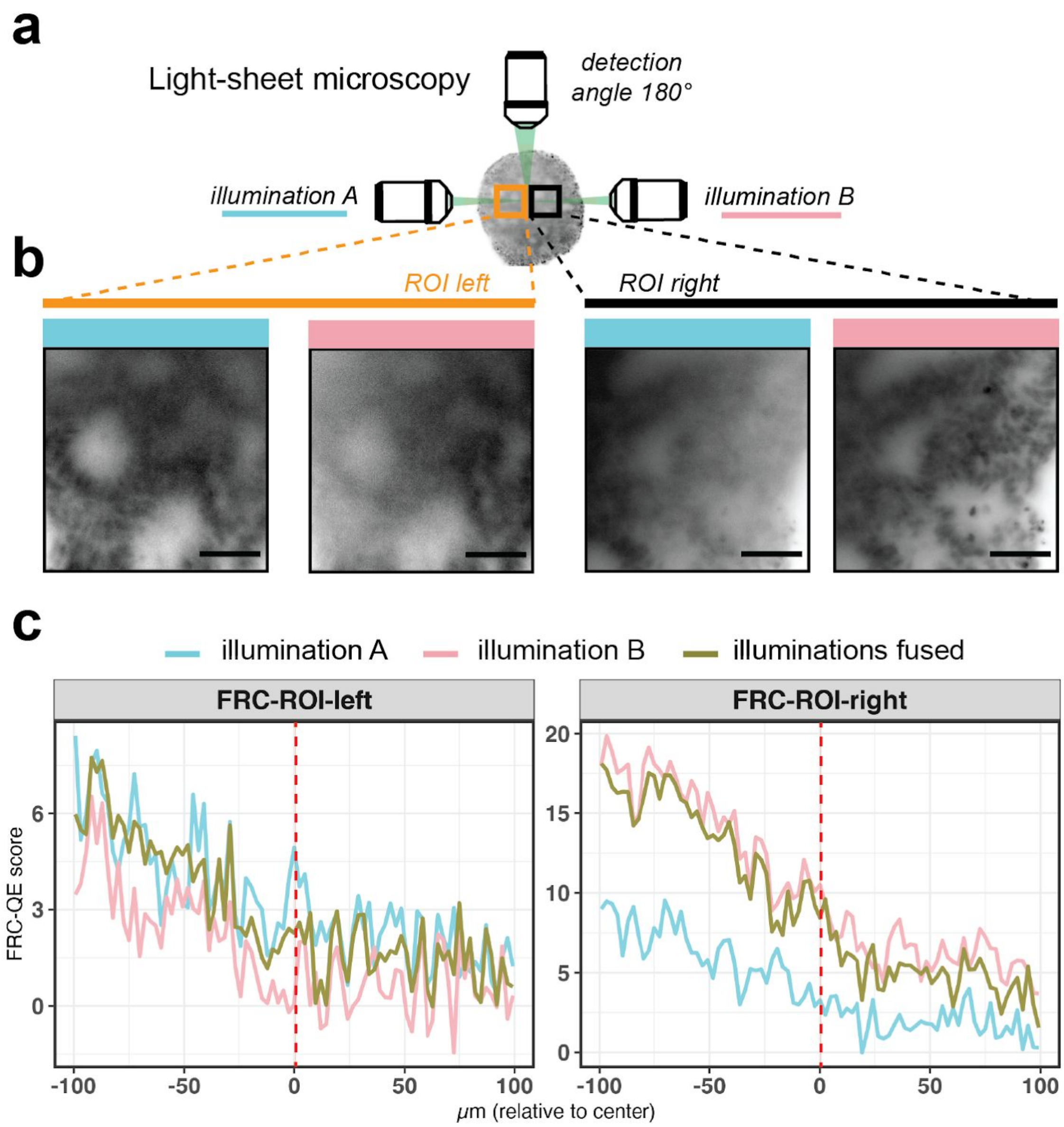

Supplementary Figure S4: Multi-view quality estimation - dual illumination. (a) Schematic of multi-view light-sheet microscopy with dual-view illumination. (b) Depicts example images for each of the two illuminations (unfused) at two different positions within the organoid. (c) Represents FRC-QE scores for unfused as well as fused images at both positions around the center of the organoid. Dotted red lines correspond to the corresponding panels above as indicated. Brightness and contrast was adjusted individually for the example images.

[Supplementary Figure S5]

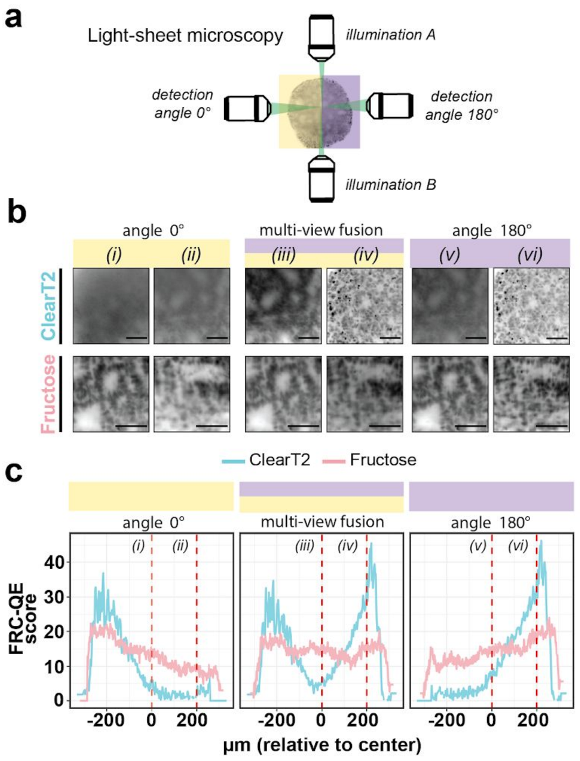

Supplementary Figure S5: Multi-view quality estimation - different angles. (a) Schematic of multi-view light-sheet microscopy with dual-view illumination and acquisition from two angles. (b) Depicts example images of multi-view fused organoid data as well as images coming only from either angle 0° or angle 180° respectively (in all cases illumination A and illumination B were fused). (c) Represents the corresponding FRC-QE score across the z-axis of the organoid. Scale bars correspond to 50 µm. Dotted red lines correspond to the corresponding panels above as indicated. Brightness and contrast was adjusted individually for the example images.

[Supplementary Figure S6]

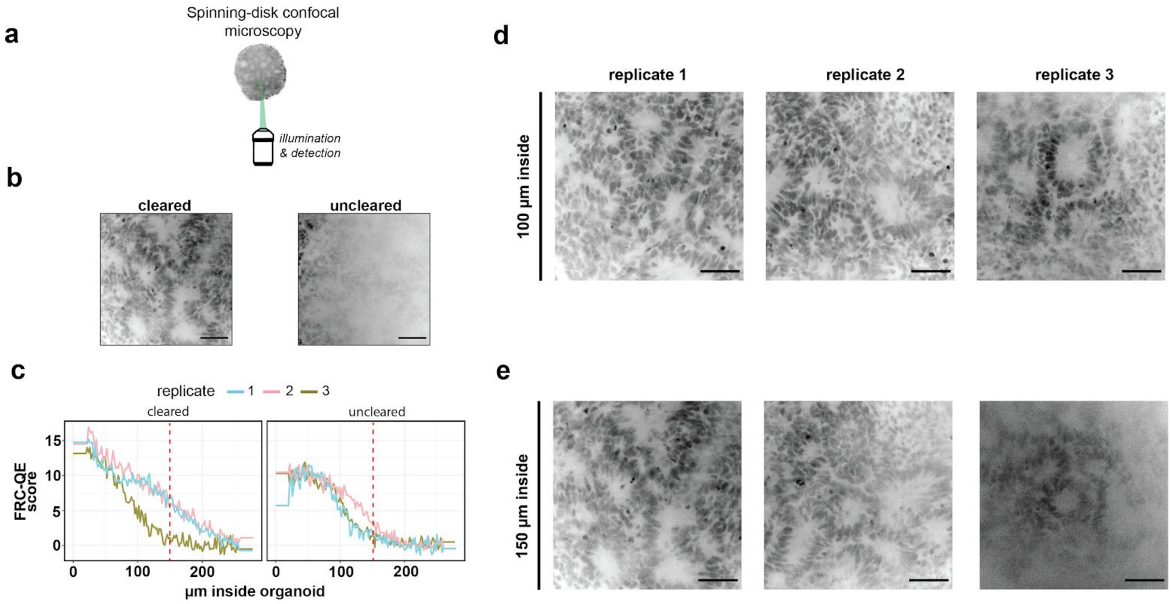

Supplementary Figure S6: FRC-QE for spinning-disk confocal microscopy data. (a) Schematic of spinning-disk confocal microscopy. (b) Two example images for Fructose-Glycerol cleared and uncleared organoids (both 150 µm inside the organoid). (c) Depicts corresponding FRC-QE score curves for 3 replicates each. (d) shows similar image quality for Draq5 signal in three replicates of Fructose cleared organoids at the surface of the organoid (50 µm). (e) Towards the center of the organoid (150 µm inside the organoid) image quality differs between replicates with replicate 3 showing lower image quality and increased light scattering. Scale bars correspond to 50 µm. Dotted red lines correspond to the corresponding panels above as indicated. Brightness and contrast was adjusted individually for the example images.

[Supplementary Figure S7]

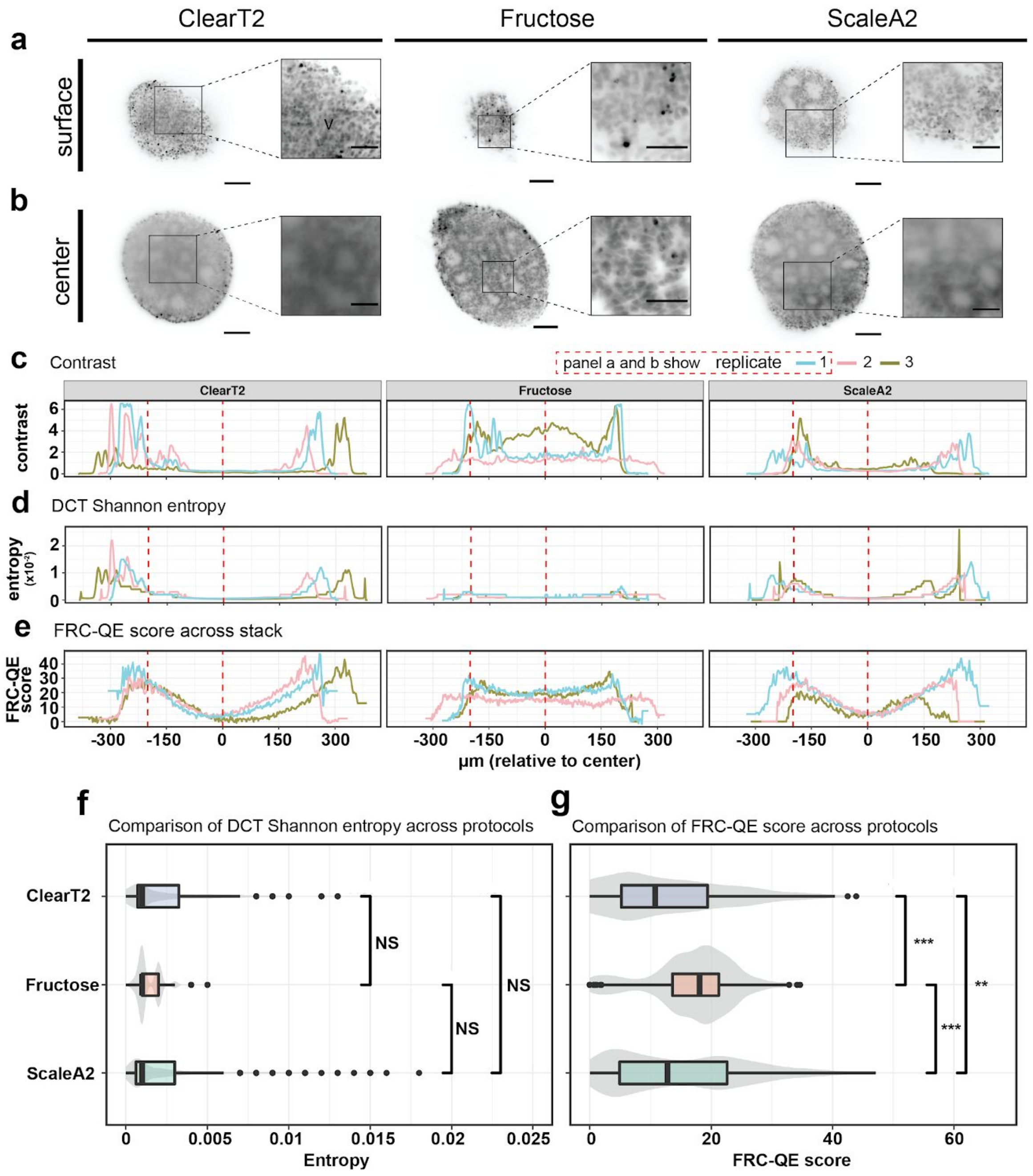

Supplementary Figure S7: Comparing image quality across protocols using FRC-QE. (a-b) Example images for each protocol, where (a) is a location at the edge of the organoid (center location minus 200 µm) and (b) corresponds to the center of the organoid. (c-e) Image quality metrics across the organoid for three replicates each, all imaged with light-sheet microscopy and multi-view reconstructed. Replicate 1 (light blue) always corresponds to the example images shown above. Fructose-Glycerol clearing showed the lowest decrease in the FRC-QE score in the center of the organoid, indicating successful clearing. (f-g) Boxplots comparing image quality estimates across protocols. Each dataset was sampled to an equal number of measured slices (600 per protocol) and the same images were analysed by DCT Shannon entropy and FRC-QE, respectively for (f) and (g).

Boxplot center line: median. Box limits: First and third quantiles. Grey shaded area: violin plot for the same dataset. Statistical significance values were calculated using Wilcoxon rank test, NS (not significant): $p > 0.05$, **: $p < 0.01$, ***: $p < 0.001$. Dotted red lines correspond to the corresponding image slices above as indicated. Scale bars correspond to 100 µm and 50 µm for large panels and inlets, respectively. Brightness and contrast was adjusted individually for the example images.

[Supplementary Figure S8]

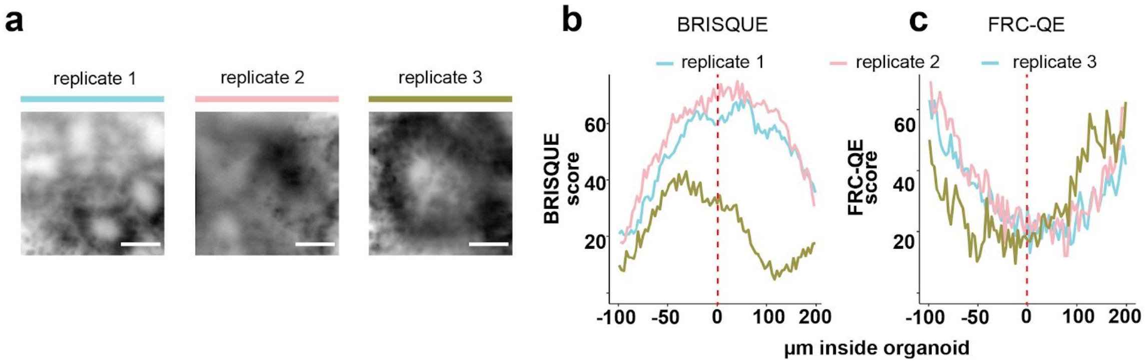

Supplementary Figure S8: Comparing FRC-QE with BRISQUE. (a) Three middle slices of organoids cleared with ScaleA2 showing similar image distortion due to insufficient clearing and light scattering. (b) shows the corresponding BRISQUE scores for image slices surrounding the center of the organoid. (c) depicts the corresponding FRC-QE scores. Note that the lower the BRISQUE score the better the image quality according to the method. Dotted red lines correspond to the position of the image slices shown in (a). Scale bars are 50 µm. For an example image stack of 496 slices (400x400 pixels) running the BRISQUE algorithm on every slice took 28 seconds in total whereas our FRC-QE method takes 26 seconds (on a 2017 MacBook Pro with 3.2 GHz and 16 GB RAM).

[Supplementary Table 1]

Imaging parameters for light-sheet imaging

Protocol	ID	replicate ID in Figure 4	x (µm)	y (µm)	z (µm)	lightsheet thickness (µm)	laser intensities	exposure time (ms)
**ClearT2**	org1	replicate 3	0.458	0.458	2.42	4.8	H: 20%, MT: 15%, D5: 15%	30
	org2	replicate 2	0.458	0.458	2.42	4.8	H: 20%, MT: 15%, D5: 15%	30
	org3	replicate 1	0.458	0.458	2.42	4.8	H: 20%, MT: 15%, D5: 15%	30
**Fructose**	org2	replicate 3	0.318	0.318	1.7	5.61	H: 20%, MT: 15%, D5: 15%	20
	org4	replicate 2	0.301	0.301	1.45	5.64	H:10%, MT: 5%, D5: 10%	30
	org5	replicate 1	0.301	0.301	1.55	6.85	H:10%, MT: 5%, D5: 10%	30
**ScaleA2**	org1	replicate 3	0.458	0.458	2.42	4.8	H: 20%, MT: 15%, D5: 15%	30
	org2	replicate 1	0.458	0.458	2.42	4.8	H: 20%, MT: 15%, D5: 15%	30
	org3	replicate 2	0.458	0.458	2.42	4.8	H: 20%, MT: 15%, D5: 15%	30

Supplementary Material and Methods

Cell Culture and generation of human cerebral organoids

Cerebral organoids were generated from the human induced pluripotent stem cell (hiPSC) line BIHi005-A (https://hpscreg.eu/cell-line/BIHi005-A). HiPSC line identity and integrity was verified at regular intervals. hiPSC were cultured in E8 medium in Geltrex-coated (Thermo Fisher) culture plates. For cerebral organoid induction, after single cell passaging hiPSC were first placed in neural induction medium (NIM, DMEM/F-12 (Thermo Fisher), 2.5 mM glutamine (Thermo Fisher), 15 mM HEPES (Thermo Fisher), 1x B27 (Thermo Fisher), 1x N2 (Thermo Fisher), 2 µM Dorsomorphin (Biovision), 10 µM SB431542 (Reagents Direct), 100 U/ml penicillin, 100 µg/ml streptomycin (Thermo Fisher)) for 6 days with daily medium changes. Next, medium was changed to neural expansion medium (NEM, 0.5x Neurobasal medium (Thermo Fisher), 0.5x Advanced DMEM/F12 (Thermo Fisher), 1x Neural Induction supplement (Thermo Fisher), 5 µM Y-27632 (Wako), 100 U/ml penicillin, 100 µg/ml streptomycin (Thermo Fisher)). Then, 7000 cells per well were seeded in a 96 well ultra low attachment round bottom plate (Corning) in neural expansion medium (NEM) and centrifuged (300x g, 5 minutes). Medium was changed to neural medium (NM, Neurobasal medium (Thermo Fisher), 1x B27 (Thermo Fisher), 2 mM Glutamax (Thermo Fisher), 20 ng/ml rhEGF (Peprotech), 20 ng/ml rhFGF-basic 154 a.a. (Peprotech), 100 U/ml penicillin, 100 µg/ml streptomycin (Thermo Fisher)), and replaced daily until day 4 and every other day thereafter. On day 6, medium was changed to neural differentiation medium (NDM, Neurobasal medium (Thermo Fisher), 1x B27 (Thermo Fisher), 2 mM Glutamax (Thermo Fisher), 20 ng/ml rhNT3 (Peprotech), 20 ng/ml rhBDNF (Peprotech), 100 U/ml penicillin, 100 µg/ml streptomycin (Thermo Fisher)). NDM was replaced every other day. Cerebral organoids were harvested on day 37.

Sample clearing

For clearing, we used brain organoids of approximately 600 µm diameter. The organoids were stained with 5 µM Draq5, 5 µM Hoechst 33342 and 250 nM MitoTracker Red CMXRos (Thermo M7512) in NDM for 30 min at 37°C, fixed with 4% paraformaldehyde (PFA) for 30 min at room temperature, washed three times in PBS and stored in PBS. Clearing was performed based on three published clearing methods: ClearT2[1], ScaleA2[2] and Fructose-Glycerol[3] that were carried out according to the published protocols. Briefly, for the ClearT2 protocol, fixed organoids were incubated for 10 min at RT in a solution of 25% formamide/10% polyethylene glycol (PEG), followed by a 5 min incubation in a 50%

formamide/20% PEG solution. Finally, organoids were immersed in fresh 50% formamide/20% PEG and incubated for 60 min at RT. All steps were carried out under gentle movement. In the ScaleA2 protocol, scale clearing solution consisted of 4 M urea, 0.1% wt/vol Triton X-100, and 10% wt/wt glycerol in water. Fixed organoids were incubated for 24h in fresh Scale clearing solution at room temperature and the solution was changed twice every 24 hours until 3 days. For the Fructose-glycerol solution, fixed organoids were placed on a heat block at 40°C, then resuspended in a 60% (vol/vol) glycerol and 2.5 M fructose/4% low melting point agarose solution. For light-sheet imaging, the mix was aspirated with a glass capillary and solidified at 4°C. Samples were incubated for 24h in the capillary before imaging. For spinning-disk confocal microscopy, fixed organoids were incubated for 20 minutes in fructose-glycerol clearing solution (60% (vol/vol) glycerol and 2.5M fructose) or PBS (negative control) before mounting.

Light-sheet microscopy

For light-sheet imaging, cleared organoids were embedded in 2% low melting point agarose columns using glass capillaries (Zeiss). The light-sheet microscope used was a commercial Zeiss light-sheet Z.1 microscope. Glass capillaries were inserted into the imaging chamber of the microscope and the agarose column was extruded into the chamber filled with imaging solution. Chambers containing the organoids cleared by CleartT2 and ScaleA2 protocols were filled with water. For fructose-glycerol cleared organoids the imaging chamber was filled with fructose–glycerol clearing solution and we allowed the sample to settle in the imaging chamber overnight to improve sample clearing. Images were acquired using 10× illumination objectives and a 20× detection objective. Draq5 was imaged with the 639 nm laser line. Laser power and microscope parameters are indicated in Supplementary table 1. Each organoid was imaged from two opposing angles, each with two illuminations, resulting in 4 views for each organoid. Multi-view reconstruction was performed in BigStitcher as previously described[4]. Briefly, interest point-detection was performed on cell nuclei (Draq5 staining) for each view. Next, the 4 views were registered by the descriptor-based translation-invariant algorithm. Fused images were exported as TIFF files.

Spinning-disk confocal microscopy

For spinning disk confocal microscopy, cleared organoids were placed in µ-Slide 8 Well chamberslides (Ibidi, 80827) and attached with one drop of 4% low melting point agarose. Imaging was performed on a PerkinElmer Opera Phenix with a 20x water objective (NA=1.0) in spinning-disk confocal mode, controlled by Harmony v 4 software. Lateral resolution for all

images was 0.3 µm with 2 µm spacing between z-slices. Laser power and exposure time were kept constant for all samples.

FRC-QE

Fourier Ring Correlation (FRC) was developed to measure image resolution based on two independent realizations of the same image, i.e. the same image taken twice.[5,6] After performing a Fast Fourier Transform of both images, FRC computes independent correlations for each frequency band (all circles with an integer radius in Fourier Space), where high frequencies represent high resolution data and lower frequencies represent lower resolution data in the original images. High correlation for a certain frequency band confirms that the same image information is present at that resolution, whereas low correlation points to noise being the dominant factor. Typically correlation is high for low frequencies and is decreasing with higher frequencies. FRC defines correlation threshold values in that frequency-vs-correlation plot, which define the actual image resolution for each pair of images analyzed. Two limitations of FRC are that (1) it requires two independent images, i.e. dedicated acquisitions need to be performed in order to use FRC and (2) it is sensitive to stationary high-frequency noise (e.g. patterned camera noise) that induces correlations at high frequencies.

FRC-QE is based on the relative Fourier ring correlation (rFRC) that we previously[4] developed, which is an extension of the FRC that can be computed from single images and is insensitive to static high-frequency noise patterns. We take advantage of the fact that consecutive image planes along the z-axis are very similar due to the axial extent of the PSF. Hence, computing the FRC values between two z-slices and integrating them over all frequencies yields a robust quality metric, with low score indicating low image quality. However, here we found that using z-slices adjacent to each other can result in overall too high correlation in some areas of some image stacks. We hypothesize that inaccurate movement of the acquisition stage in z might be responsible. Instead, we therefore compute the FRC for slice z using the slices z+1 and z-1, which yields a more robust FRC readout (Supplementary Figure S1).

To exclude artifacts caused by nonspecific patterned noise or imaging artifacts (e.g. induced by camera noise) leading to increased correlation at higher frequencies we calculate the relative FRC (rFRC) by subtracting a smoothed FRC baseline of z-slices spaced by $z+m$ and $z-m$ (default m=10) slices that are beyond the axial extent of the point spread function (PSF).

The integral over the subtracted curves yields the FRC-QE score. FRC-QE does not represent an actual measure of image resolution. Instead it describes how much more image correlation by frequency there is at the location where it is computed, as compared slices that are out of range of the PSF. Naturally, the actual values of the FRC-QE score depend significantly on the z-step size, type of content (e.g. nuclei stain), the PSF size and the FFT size in which the FRC is computed. It is therefore important to keep these parameters constant when comparing outcomes of different experiments.

We implemented the adapted rFRC calculation at defined block sizes for the FRC-QE Fiji plugin. For the light-sheet microscopy data shown here, we used a 400x400 pixel window (spanning all z-slices) as input for each organoid to compute the FRC-QE score using a 200x200 pixel block, resulting in 4 subtiles spanning the 400x400px window, each corresponding to a distinct FRC-QE score for each plane. For each plane, we take the median value of the 4 subtiles yielding the final FRC-QE score. Thus, by taking the median value of 4 spatially separated tiles, high frequency correlations caused by imaging artifacts are suppressed.

Comparison to other image quality metrics

Conceptually, FRC-QE is different from other methods like DCT Shannon entropy (a simple no reference image quality algorithm (NR-IQA)) because its measurements are more abstract. DCT Shannon entropy measures the information content of the frequency space. However, noise also produces high frequency components and DCT Shannon entropy is therefore not able to differentiate between noise and content. DCT Shannon entropy nevertheless works robustly on single stacks, which is required for autofocussing, since noise patterns typically do not change locally. FRC-QE on the other hand measures the correlation in between individual frequencies relative to a background correlation and is thereby able to differentiate noise from actual image content. Furthermore, it is able to ignore artifacts such as camera noise or local dirt. This allows for a higher degree of invariance and thereby enables the comparison of different clearing protocols for example.

Furthermore, FRC-QE represents a new NR-IQA algorithm that is adapted to the challenges of fluorescence microscopy (e.g. normalizing for camera noise), which enables more robust comparison across replicates and protocols. We have also tested its performance in comparison to the dubbed blind/referenceless image spatial quality evaluator (BRISQUE) algorithm, a state-of-the-art reference-based IQA[7]. While BRISQUE overall showed comparable performance to FRC-QE, we note inconsistencies when estimating the image quality of the center region in insufficiently cleared organoids, where images of similar quality differ significantly in their score (Supplementary Figure S8). It is important to note that

BRISQUE works by comparing image features to features extracted from images of annotated datasets with known distortions. Since these datasets usually do not contain fluorescence microscopy images, distortions present in our data might be missing in the corresponding training data for BRISQUE, potentially resulting in a less reliable image quality estimation as previously observed[8]. Since appropriate training data would depend on protocol type, fluorescent staining and chosen microscope parameters, we believe that FRC-QE as a NR-IQA method should enable more straightforward automated quality estimation compared to a reference-based IQA.

Contrast was calculated for each plane as minimum pixel intensity subtracted from maximum pixel intensity. All intensity and contrast values were divided by 10^4 for better readability. Normalized discrete cosine transform (DCT) Shannon entropy was imported and called as previously described[9]. BRISQUE scores were computed using the pybrisque package in python (https://github.com/bukalapak/pybrisque).

Statistical analysis and visualization

Statistical analysis and visualization of the data was done in R (Version 3.6.1), using ggplot[10] and the dplyr[11] package.

References

1. Kuwajima, T. *et al.* ClearT: A detergent- and solvent-free clearing method for neuronal and non-neuronal tissue. *Dev. Camb.* **140**, 1364–1368 (2013).
2. Hama, H. *et al.* Scale: A chemical approach for fluorescence imaging and reconstruction of transparent mouse brain. *Nat. Neurosci.* **14**, 1481–1488 (2011).
3. Dekkers, J. F. *et al.* High-resolution 3D imaging of fixed and cleared organoids. *Nat. Protoc.* (2019) doi:10.1038/s41596-019-0160-8.
4. Hörl, D. *et al.* BigStitcher: reconstructing high-resolution image datasets of cleared and expanded samples. *Nat. Methods* (2019) doi:10.1038/s41592-019-0501-0.
5. Saxton, W. O. & Baumeister, W. The correlation averaging of a regularly arranged bacterial cell envelope protein. *J. Microsc.* **127**, 127–138 (1982).
6. Heel, M. V. Similarity measures between images. *Ultramicroscopy* **21**, 95–100 (1987).

7. Mittal, A., Moorthy, A. K. & Bovik, A. C. No-Reference Image Quality Assessment in the Spatial Domain. *IEEE Trans. Image Process.* **21**, 4695–4708 (2012).

8. Koho, S., Fazeli, E., Eriksson, J. E. & Hänninen, P. E. Image Quality Ranking Method for Microscopy. *Sci. Rep.* **6**, 28962 (2016).

9. Royer, L. A. *et al.* Adaptive light-sheet microscopy for long-term, high-resolution imaging in living organisms. *Nat. Biotechnol.* **34**, 1267–1278 (2016).

10. Wickham, H. *ggplot2: Elegant Graphics for Data Analysis*. (Springer-Verlag New York, 2016).

11. Wickham, H., François, R., Henry, L. & Müller, K. *dplyr: A Grammar of Data Manipulation*. (2018).
